# MID1 Catalyzes the Ubiquitination of Protein Phosphatase 2A and Mutations within Its Bbox1 Domain Disrupt Polyubiquitination of Alpha4 but Not of PP2Ac

**DOI:** 10.1371/journal.pone.0107428

**Published:** 2014-09-10

**Authors:** Haijuan Du, Kuanlin Wu, Alma Didoronkute, Marcus V. A. Levy, Nimish Todi, Anna Shchelokova, Michael A. Massiah

**Affiliations:** Department of Chemistry, George Washington University, Washington, District of Columbia, United States of America; Loyola University Chicago, Stritch School of Medicine, United States of America

## Abstract

MID1 is a microtubule-associated protein that belongs to the TRIM family. MID1 functions as an ubiquitin E3 ligase, and recently was shown to catalyze the polyubiquitination of, alpha4, a protein regulator of protein phosphatase 2A (PP2A). It has been hypothesized that MID1 regulates PP2A, requiring the intermediary interaction with alpha4. Here we report that MID1 catalyzes the *in* *vitro* ubiquitination of the catalytic subunit of PP2A (PP2Ac) in the absence of alpha4. In the presence of alpha4, the level of PP2Ac ubiquitination is reduced. Using the MID1 RING-Bbox1-Bbox2 (RB1B2) construct containing the E3 ligase domains, we investigate the functional effects of mutations within the Bbox domains that are identified in patients with X-linked Opitz G syndrome (XLOS). The RB1B2 proteins harboring the C142S, C145T, A130V/T mutations within the Bbox1 domain and C195F mutation within the Bbox2 domain maintain auto-polyubiquitination activity. Qualitatively, the RB1B2 proteins containing these mutations are able to catalyze the ubiquitination of PP2Ac. In contrast, the RB1B2 proteins with mutations within the Bbox1 domain are unable to catalyze the polyubiquitination of alpha4. These results suggest that unregulated alpha4 may be the direct consequence of these natural mutations in the Bbox1 domain of MID1, and hence alpha4 could play a greater role to account for the increased amount of PP2A observed in XLOS-derived fibroblasts.

## Introduction

**Figure 1:**
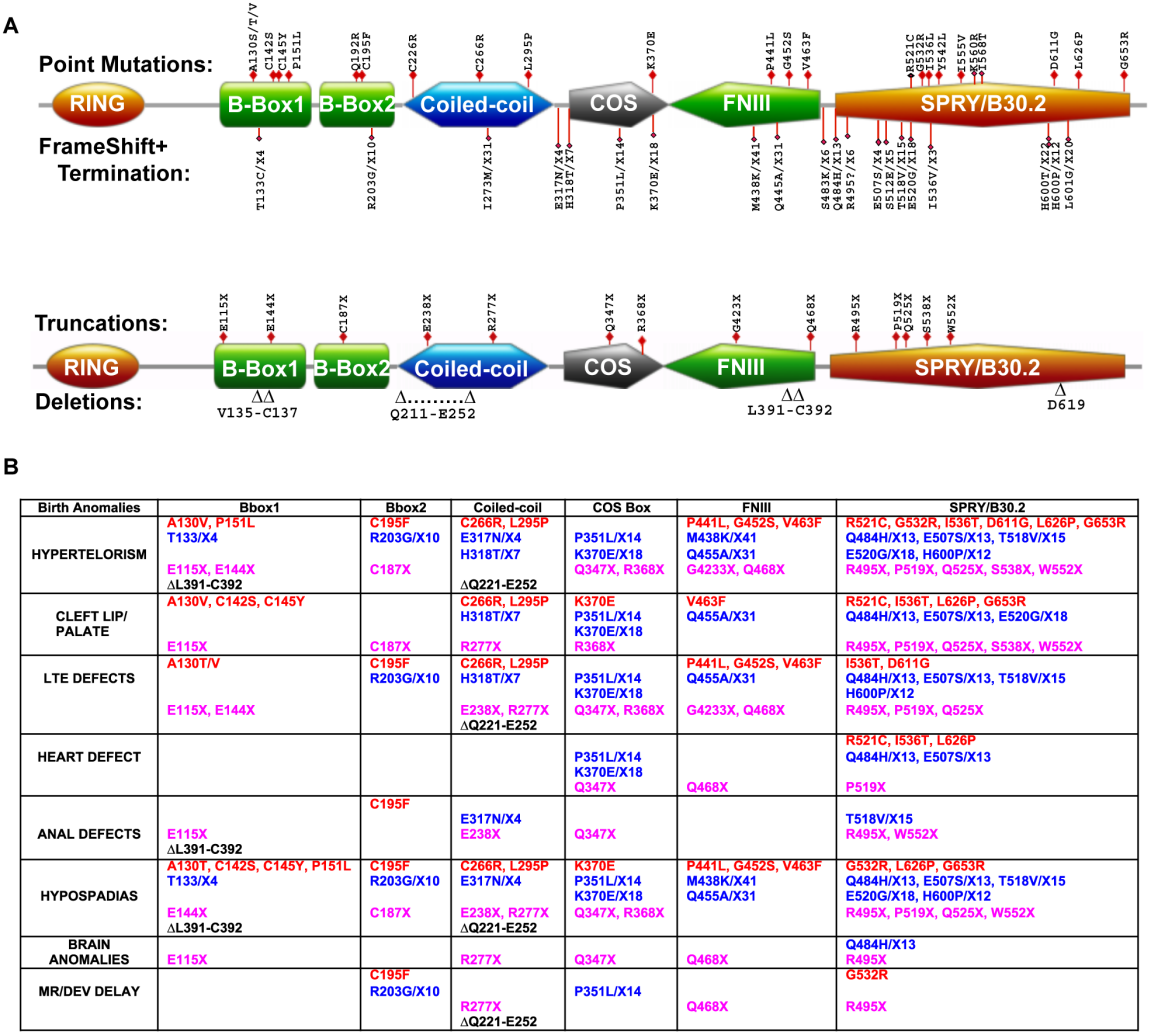
Natural mutations of MID1 and birth anomalies. **A.** Schematic representation of MID1 showing the locations of the various types of mutations. MID1 is a member of the TRIM family of proteins, consisting of seven distinct domains. The RING and Bbox domains coordinate two zinc ions each and adopt ββα-RING folds. The coiled-coil (CC) domain is important for MID1 dimerization. The CC contains a region that is required for microtubule association, denoted as the COS box [35]. The C-terminal half contains the fibronectin type III (FNIII) and SPRY/B30.2 domains. Superimposed on the schematic structure are the four types natural mutations observed with patients with X-linked Opitz G Syndrome. **B.** Summary of all reported mutations, to date, of MID1 and the associated anomalies [27], [28], [29], [30], [31], [32], [33], [34]. The four types of mutations observed are color-coded (red = point mutation, blue = frame shifts, magenta = termination/truncation, and black = deletion). Each column lists the different types of mutations (color coded) associated with each domain and grouped with the reported birth defects. X indicates a truncation mutation. The most common defects are hypertelorism, hyperspadias and cleft lip/palate based on the number of mutations associated with these defects. Other craniofacial anomalies that are not listed included low set ears, widows peak, heart defects and small jaw or chin to name a few.

MID1 is a member of the tripartite motif (TRIM) protein family consisting of the N-terminal RING, two B-box, and Coiled-Coil (RBCC) domains (Figure 1A). The RING and Bbox are the domains that possess E3 ligase functionality and important for substrate specificity [1], [2]. Recently, we demonstrate that the MID1 Bbox domains are essential for optimal MID1 E3 ligase functionality [1], [2]. The MID1 Bbox domains exhibit weaker E3 ligase compared the RING domain, as evident by autoubiquitination activities. When the RING domain is natively linked to Bbox1 or both Bbox domains, the rate of E3 ligase activity is increased by at least 10-fold, suggesting that the Bbox domains may have, in part, E4 ligase functionality [1], [2].

The Bbox domains, which coordinate two zinc-ions and adopt ββα-RING folds, are required for the efficient ubiquitination of alpha4, an oncogenic protein initially identified as an immunoglobin binding protein (IgBP-1). MID1 RING domain catalyzes only the monoubiquitination of alpha4, but the RING in tandem with Bbox1 or both Bbox domains (RING-Bbox1 and RING-Bbox1-Bbox2) catalyzes the polyubiquitination of alpha4. Neither the individual nor the tandem Bbox domains catalyzes the polyubiquitination of alpha4.

Alpha4 down-regulates protein phosphatase 2A (PP2A) in numerous cellular pathways, including the target of rapamycin (TOR) pathway [3], [4], [5]. PP2A is a major serine/threonine phosphatase that regulates cellular processes associated with metabolism, cell-cycle progression, and apoptosis [6], [7], [8], [9], [10], [11], [12], [13], [14], [15], [16], [17]. PP2A is a heterotrimeric complex consisting of a catalytic subunit (PP2Ac), a scaffolding (PP2A/a, PR65) subunit, and numerous regulatory (PP2A/b) subunits [9], [18], [19], [20]. Alpha4 binds the PP2Ac subunit and displaces the scaffold and regulatory subunits [5], [21]. Alpha4 can sequester PP2Ac in a stable reserve, supposedly protecting it from degradation, and releasing it to counter stressful stimuli to the cell [4], [22].

The Bbox1 domain binds alpha4 and this interaction is hypothesized to recruit PP2Ac to MID1 for ubiquitin-mediated degradation [17], [23], [24], [25]. XLOS-derived fibroblast cells show an increased cellular PP2Ac concentration, suggesting that MID1 and alpha4 are important for PP2Ac homeostasis [17], [24], [25], [26]. However, to date, there has not been any evidence that MID1 directly targets PP2Ac for ubiquitin-mediated degradation.

In the past decade, there have been numerous reports identifying mutations of MID1 from individuals with ventral midline anomalies that affect the brain, face, heart and genitalia (Figure 1B) [27], [28], [29], [30], [31], [32], [33], [34]. Individuals with these defects are diagnosed with X-linked Opitz G Syndrome (XLOS). The MID1 protein from XLOS patients is observed to have point mutations, frame-shifts, deletions, and truncations in all its domains except for the region encompassing the RING domain (Figure 1). Despite the diversity of mutations, there is no pattern or correlation of birth defects associated with specific mutations. The most common anomalies include hypertelorism, hypospadias, clefts of the lip and/or palate (CL/P), and laryngotracheoesophageal (LTE) cleft (Figure 1B). These anomalies are associated with mutations within the MID1 Bbox domains.

*In vivo* fluorescence studies using MID fused to a green fluorescent protein tag (GFP-MID1) reveal that a number of XLOS-observed mutations within the C-terminal half of MID1 cause MID1 to form cytoplasmic ‘clumps’ instead of normally being associated with the microtubules [27], [35], [36], [37]. Deletion of the SPRY/B30.2 domain (MID1delCTD) results in MID1 aggregation [17], [35], [38]. The overexpression of exogenous MID1delCTD in COS-1 and MDCK cells have been shown to sequester native endogenous MID1 through aggregation and decrease the activity of native MID1 [1], [27], [35], [36], [39]. In contrast, there is no information about the effects of mutations within the N-terminal half of MID1, specifically within the Bbox domains.

In this article, we present data showing that MID1 catalyzes the ubiquitination of PP2Ac in the absence of alpha4. Using the MID1 RB1B2 protein construct, we observed that the RB1B2 proteins with natural mutations within the Bbox1 domain still catalyze the ubiquitination of PP2A but not the polyubiquitination of alpha4. These results suggest that alpha4 may play a greater role to account for the increase of PP2A observed in XLOS-derived fibroblasts.

## Methods and Materials

### Plasmid construction for recombinant protein production

The DNA encoding the MID1 RING-Bbox1-Bbox2 (RB1B2, residues 1–214) was cloned into an Expresso T7 plasmid (Lucigen Corp., Middleton, WI) containing a sequence for a C-terminal His_6_-tag. Mutations within the DNA for Bbox1 (A130S/T/V, C142T, C145S) and Bbox2 (C195F) domains were performed using standard PCR mutagenesis protocol using the RB1B2 domain construct. The His_6_-tagged alpha4 were cloned as previously described [1], [2]. The cDNA of PP2Ac was cloned from human PP2A into the pET151/D-TOPO vector with a N-terminal His_6_-tagged using the following primers: 5′CACCATGGACGAGAAGGTGTTCACC3′ and 5′TTACAGGAAGTAGTCTGGGGTACGACG3′. DNA sequencing confirmed the integrity of DNA inserts and mutations before the plasmids were transformed into BL21(*DE3*) cells.

The BL21(*DE3*) cells expressing the wild type and mutants MID1 RB1B2 constructs, alpha4, and PP2Ac were grown in LB media at 37°C to an OD_600_ of ∼0.5 and induced with 0.2 mM isopropyl β-D-1-thiogalactopyranoside for 18 hr at 18°C. The RB1B2 protein and the various mutants were purified as previously described [1], [2]. Briefly, harvested cells were stored at −80°C before being resuspended in buffer 50 mM Tris, 200 mM NaCl (pH 7.5), with protease inhibitors and lysed with sarkosyl and sonication. Lysates were clarified by centrifugation at 20,000×g at 4°C for 30 min. The RB1B2, alpha4, and PP2Ac proteins were purified using standard the Ni^2+^-NTA affinity chromatography approach.

### In vitro ubiquitination assay using purified recombinant proteins

Human Ub-activating enzyme E1, human Ub-conjugating enzymes (UbcH5b), UbcH (E2) enzyme, and biotinylated ubiquitin were purchased from BostonBiochem (Bio-Techne Inc., Minneapolis, MN). The autoubiquitination assays of the MID1 E3 ligase domains were performed in a total volume of 30 µL. The mixture contained 1 unit (generates 1 µmol of phosphate per minute) of thermostable inorganic pyrophosphatase (New England Biolabs), 1 mM DTT, 5 mM ATP-Mg^2+^, 0.125 µM E1, 2.5 µM E2 (UbcH5b/UBE2D2), and 2 µM biotinylated ubiquitin (Ub) in 20 mM Tris–HCl (pH 7.5), and purified recombinant proteins. The ubiquitination assays were incubated at 30°C for 90 minutes and the reaction terminated with 2x SDS-PAGE non-reducing sample loading buffer. The ubiquitination of alpha4 and PP2Ac were performed similarly to the autoubiquitination reaction by adding these proteins to the reaction mixture and using non-biotinylated Ub. The reactions were performed at 30°C for 4 hrs. The reaction products were subsequently evaluated by SDS-PAGE and chemiluminescent western blot (WB) analysis. For the autoubiquitination assay, the antibody used was avidin, which is specific for biotin. For the assays containing the alpha4 and PP2Ac proteins, the antibodies were specific for the C-terminal region of alpha4 (cat #sc-32174) and PP2Ac (cat #sc-376673) and were purchased from Santa Cruz Biotechnology. Control reactions were performed by omitting individual components from the assay. WB digital images were acquired with a Syngene G5:Box chemidoc system.

## Results

### MID1 catalyzed the ubiquitination of protein phosphatase 2A catalytic subunit

To understand how mutations within the Bbox domains of MID1 might affect the regulation of PP2Ac, we began by determining whether MID1 could directly catalyze the ubiquitination of PP2Ac. To this end, we performed *in* *vitro* ubiquitination studies using recombinant full-length MID1 and PP2Ac. MID1 was constructed with a maltose-binding protein (MBP) tag at its N-terminus to enhance solubility.

**Figure 2:**
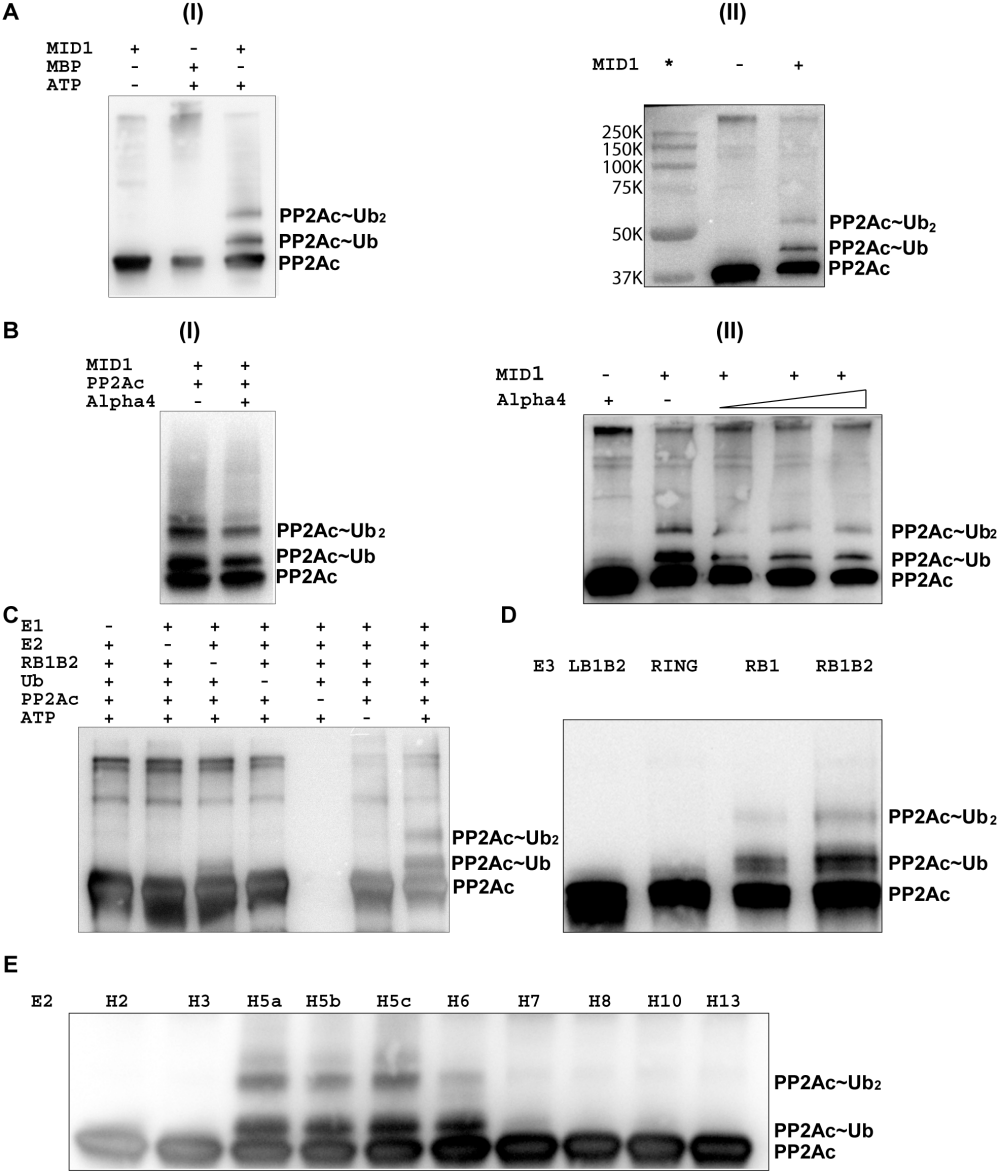
MID1 catalyzed the ubiquitination of PP2Ac. **A.** Western-blot (WB) showing *in* *vitro* ubiquitination of PP2Ac by MBP-tagged full-length MID1. (1) Lanes 1 and 2 show the results of ubiquitination assay for control reactions. In lane 1 ATP was omitted (no reaction) and in lane 2, only MBP was used. Lane 3 contains the results for the MPB-tagged MID1-catalyzed reaction. Two strong shifted bands corresponding to mono- and di-ubiquitinated PP2Ac and a faint smearing pattern indicative of poly-ubiquitinated PP2Ac were observed. (ii) Lane 1 shows the Biorad visible marker and lane 2 shows a control reaction in which MBP-MID1 was omitted. Lane 3 shows the ubiquitinated PP2Ac to confirm the molecular weights of the mono- and di-ubiquitinated products. **B.** (i) Ubiquitination of PP2Ac by MBP-tagged MID1 in the absence and presence of full-length alpha4. (ii) Ubiquitination of PP2Ac with increasing amounts of alpha4. The amounts of alpha4 were present at 1:1, 1:2 and 1:4 molar ratios with PP2Ac. **C.** Ubiquitination of PP2Ac by the MID1 RB1B2 protein (RING-Bbox1-Bbox2, MID1 residues 1–214). Lanes 1–6 show the results of control experiments in which a specific component of the assay was omitted, including the E1 activating enzyme (E1) (lane 1). Mono- and di-ubiquitinated PP2Ac were observed in lane 7, with all components of the reaction present. Background noise precludes detection of polyubiquitinated products. Antibody was specific for PP2Ac. **D.** Ubiquitination of PP2Ac by various constructs of the three N-terminal domains. These domains were expressed and purified similarly, and used at roughly the same concentration for the assays. Lane 1 uses the Bbox1-Bbox2 construct that includes the amino acids from the linker region between the RING and Bbox1 domains (LB1B2, residues 71–115). Lane 2 uses a RING domain (residues 1–92) that also includes a large position of the linker region. The RB1 and RB1B2 constructs consist of residues 1–164 and 1–214 respectively. **E.** Ubiquitination of PP2Ac in the presence of 10 different E2 conjugating enzymes.

As shown in Figure 2A(i, ii), the full-length MID1 protein catalyzed the mono- and di-ubiquitination of PP2Ac as predominant products. The western blot also revealed the presence of a weak intensity smearing pattern of high-molecular weight products indicative of polyubiquitinated PP2Ac (Figure 2A(i)). As part of the control, omission of ATP and MID1 resulted in loss of the mono-, di- and polyubiquitinated PP2Ac bands, confirming that MID1 catalyzes the ubiquitination of PP2Ac (Figure 2A). The intensity of the mono-ubiquitinated PP2Ac band was greater than that of the di-ubiquitinated band, suggesting that Ub addition PP2Ac may be sequential.

### Alpha4 down-regulated the level of PP2Ac ubiquitination

The general consensus in the literature is that alpha4, which interacts with PP2Ac and MID1, is required to recruit PP2Ac to the MID1 [5], [15], [17], [40], and therefore may be necessary for efficient ubiquitination of PP2Ac. To test this hypothesis, we performed the ubiquitination assays of PP2Ac in the absence and presence of alpha4 (Figure 2B(i)). The level of PP2Ac ubiquitination was substantially less in the presence of alpha4, as evidence by the decrease of intensities of the mono- and di-ubiquitinated PP2Ac bands (Figure 2B(i, ii)). Given that previous findings suggested that alpha4 was involved in the recruiting of PP2Ac [5], [17], [21], [24], we tested whether targeting of PP2Ac might have a diphasic dependence on alpha4 concentration [41]. We anticipated that the level of ubiquitination of PP2Ac might increase as alpha4 increases but at some point the level should decrease due to competitive binding of the two proteins with MID1. We performed a series of ubiquitination assays, each with increasing amounts of alpha4 compared to PP2Ac. The concentrations of all components of the assays, except alpha4, were kept constant. When the concentrations of alpha4 were less than that of PP2Ac, we did not observe any discernable difference in the intensities of the mono- and di-ubiquitinated PP2Ac bands compared to when alpha4 was absent. At concentrations in which alpha4 was present at equimolar or greater than PP2Ac, we observed roughly the same amount of decrease in mono- and di-ubiquitinated PP2Ac (Figure 2B(ii)).

### MID1 RB1B2 E3 domains catalyzed ubiquitination of PP2Ac

We had previously demonstrated that the E3 ligase activity of MID1 was localized to the N-terminal RING-Bbox1-Bbox2 (RB1B2) zinc-binding domains [1], [2]. To determine whether these three domains could catalyze the ubiquitination of PP2Ac, we performed the ubiquitination assay with the RB1B2 protein (MID1 residues 1–214). Similar to results obtained with full-length MID1, the RB1B2 protein catalyzed the mono- and di-ubiquitination of PP2Ac (Figure 2C). The high molecular weight smear pattern was not as obvious, suggesting that domains within the C-terminal half of MID1 may contribute to the polyubiquitination of PP2Ac.

To determine how the RING and Bbox domain contributed to targeting and catalyzing the ubiquitination of PP2Ac, we performed the ubiquitination assay with the following domain constructs: RING (MID1 residues 1–92), RING-Bbox1 (RB1, residues 1–164), RING-Bbox1-Bbox2 (residues 1–214) and Bbox1-Bbox2 (LB1B2, residues 71–214). The LB1B2 protein construct included residues from the linker region between the RING and Bbox1 domains (residues 71–110) but not the RING domain. We previously observed that these four fragments have different levels of E3 ligase activity, as part of the standard autoubiquitination assay and in their ability to catalyze the polyubiquitination of alpha4 [1], [2]. As noted, our goal was to determine if similar results would be observed for the ubiquitination PP2Ac and to identify the role of the RING and Bbox domains. These domains were expressed and purified similarly, and used at roughly the same concentration for the assays. The RING domain only facilitated mono-ubiquitination of PP2Ac. Both the RB1 and RB1B2 proteins catalyzed the mono- and di-ubiquitination of PP2Ac (Figure 2D). No ubiquitination of PP2Ac was observed with the LB1B2 protein. These observations are similar to those obtained with the ubiquitination studies of alpha4 by MID1 [1], [2].

In addition to the UbcH5b (UBE2D2) E2 enzyme, our previous studies with MID1 indicated that the RB1B2 protein could interact with multiple E2 enzymes in catalyzing its own polyubiquitination and that of alpha4 [1], [2]. We tested 12 E2 enzymes to determine whether any would be more efficient in facilitating the ubiquitination of PP2Ac. We observed that PP2Ac was ubiquitinated in the presence of UbcH5a-c and UbcH6, similar to results obtained with MID1-catalyzed ubiquitination of alpha4 (Figure 2E) [1].

### XLOS-observed MID1 mutations possessed autoubiquitination activities

To investigate how XLOS-observed mutations within the Bbox domains might affect MID1 E3 ligase functionality, we compared the autoubiquitination activity of the mutant RB1B2 constructs with that of wild type RB1B2 protein. Five mutations within the Bbox1 domain (A130T/V/S, C142S, and C145T) and one within the Bbox2 domain (C195F) were designed. The A130S mutation, which is not associated with XLOS, was intended to distinguish the role of the hydroxyl from methyl group of the Thr130 mutation. These mutant RB1B2 proteins were expressed and purified similarly as the wild type RB1B2 protein. All mutant protein constructs were soluble and stable at the experimental conditions used.

**Figure 3:**
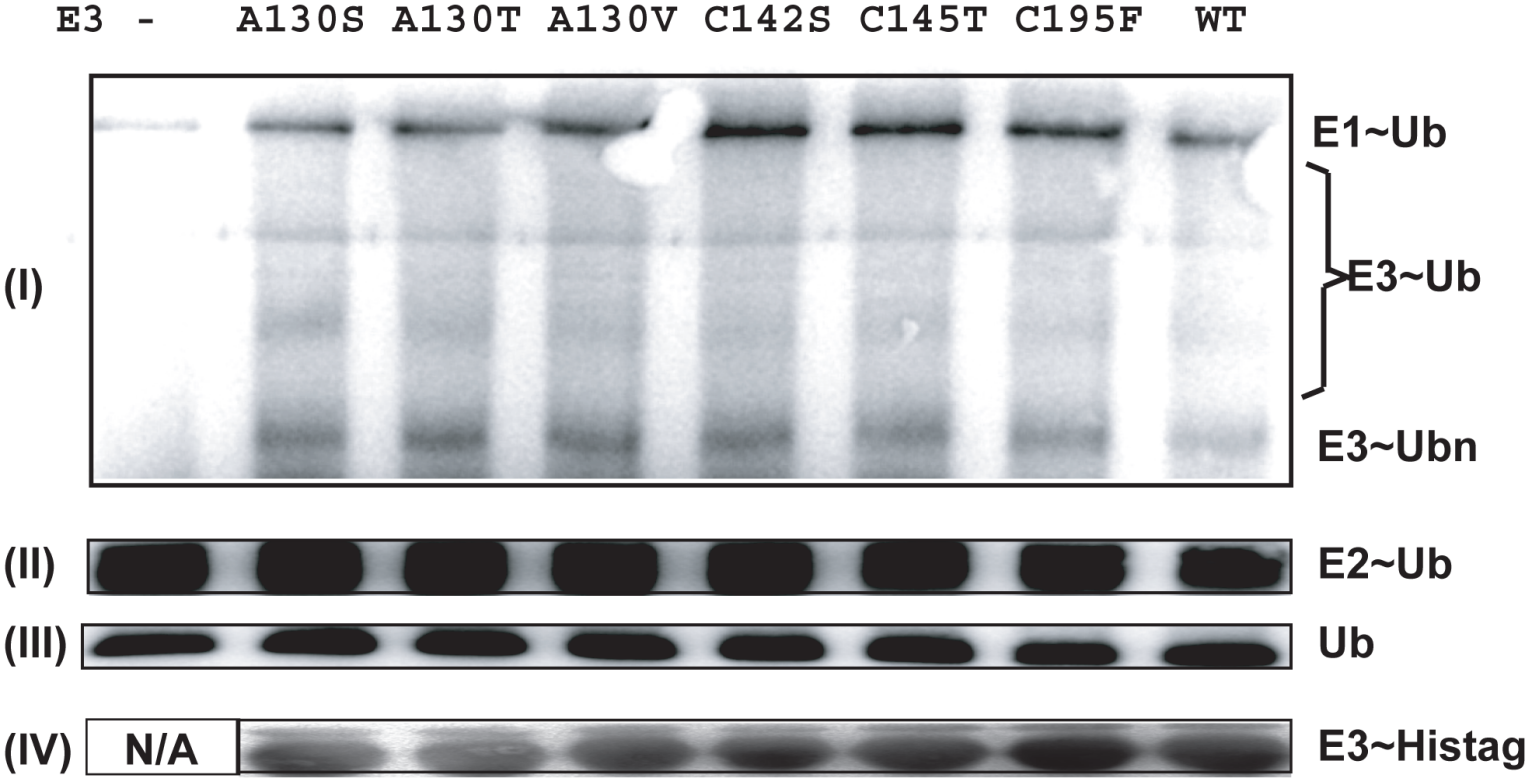
RB1B2 with mutant Bbox domains possessed auto-ubiquitination activity. (i) WB image showing the results of the E3 ligase auto-ubiquitination assay of RB1B2 harboring the C142S, C145T, C195F, and the mutation of Ala130 to Ser, Thr, and Val. Lane 1 does not contain the RB1B2 protein. (ii, iii) The WB images of the bands for the activated E2∼Ub and ubiquitin are shown separately; these bands were part of the same image as (i) but were excised due to their brightness and dynamic range problems for clarity. Antibody used with all four figures was specific for biotinylated ubiquitin. (iv) WB of the stock solutions of the various His-tagged RB1B2 constructs. No His-tagged RB1B2 protein was used in lane 1, which served as control in which the E3 was omitted. This image was taken from a separate gel because the antibody was specific for the His_6_-tag.

As shown with Figure 3, auto-polyubiquitinated products were observed with all of the RB1B2 mutant constructs, similar to the autoubiquitination activities of the wild type RB1B2 protein.

### XLOS-observed MID1 mutants catalyze PP2Ac ubiquitination

**Figure 4:**
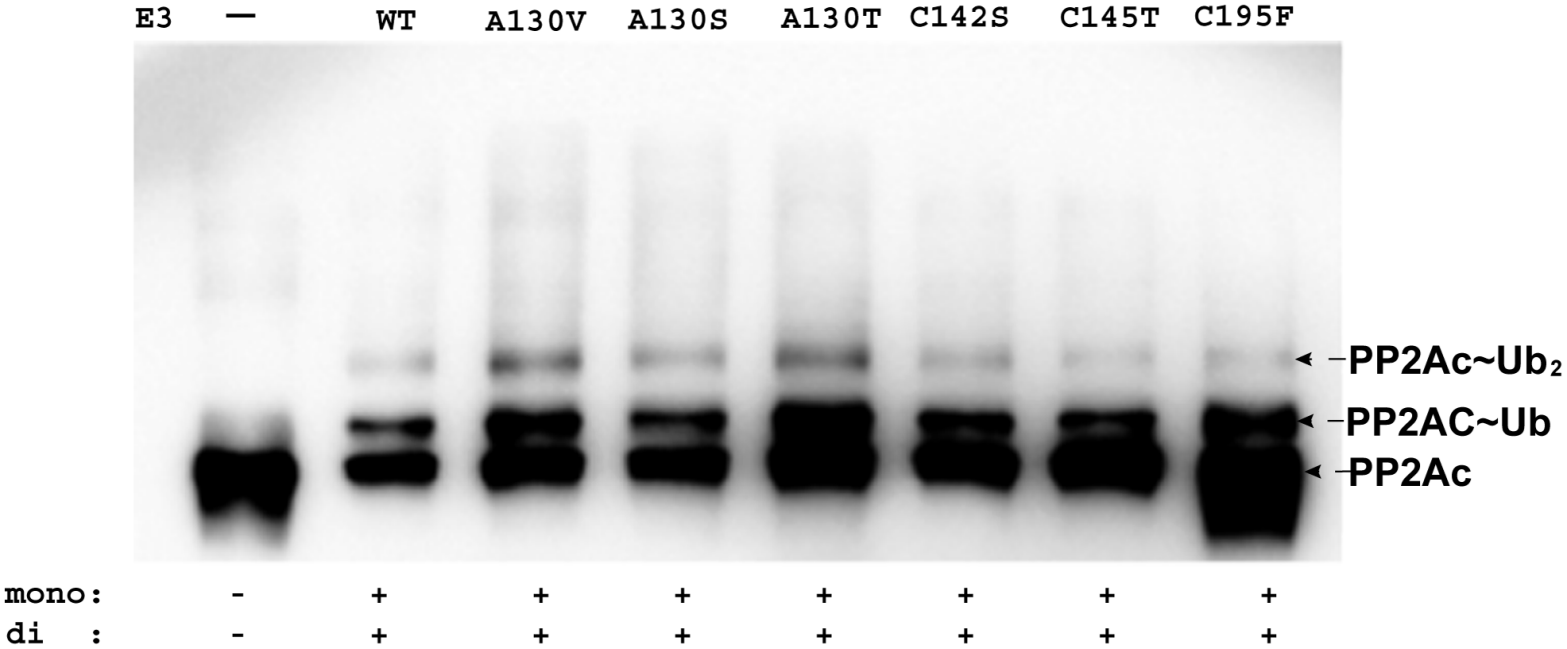
RB1B2 with mutant Bbox domains catalyzed the ubiquitination of PP2Ac. WB showing results of the ubiquitination reaction with PP2Ac using RB1B2 containing the various mutations within the Bbox domains. In lane 1, RB1B2 was omitted as control. At the bottom, the ability of the mutant RB1B2 constructs to catalyze the ubiquitination of PP2Ac is qualitatively noted. Antibody was specific against PP2Ac.

It is generally acknowledged in the literature that mutations of MID1 are associated with an increase in the amount of PP2Ac, suggesting that MID1 is involved in the regulation of PP2Ac [17], [42]. Therefore, we tested whether mutations within the Bbox domains would disrupt the ubiquitination of PP2Ac. No studies with a mutant RING domain were performed because, to date, no mutation associated with XLOS was reported for the RING domain (Figure 1). We used the RB1B2 construct because the RB1B2 mutants were easier to purify compared to full-length MID1. Moreover, we hypothesize that disruption in the mono- and di-ubiquitination of PP2Ac using the mutant RB1B2 protein would directly correlate with loss of the full-length MID1 to catalyze the ubiquitination of PP2Ac. Qualitatively, all the mutant RB1B2 constructs were observed to catalyze the mono- and di-ubiquitination of PP2Ac, similar to the wild type RB1B2 protein (Figure 4).

### XLOS-observed mutations within the Bbox1 domain abolished alpha4 polyubiquitination

In light of these results, we know that mutations within the Bbox domains are associated with physical abnormalities (Figure 1B) and therefore must have some functional effects. As noted, we recently demonstrated that MID1 catalyzed the polyubiquitination of alpha4, leading to its proteasome-mediated degradation [1]. The polyubiquitination was dependent on the interaction of the Bbox1 domain and alpha4. Thus, we anticipate that these mutations might affect the targeting of alpha4.

**Figure 5:**
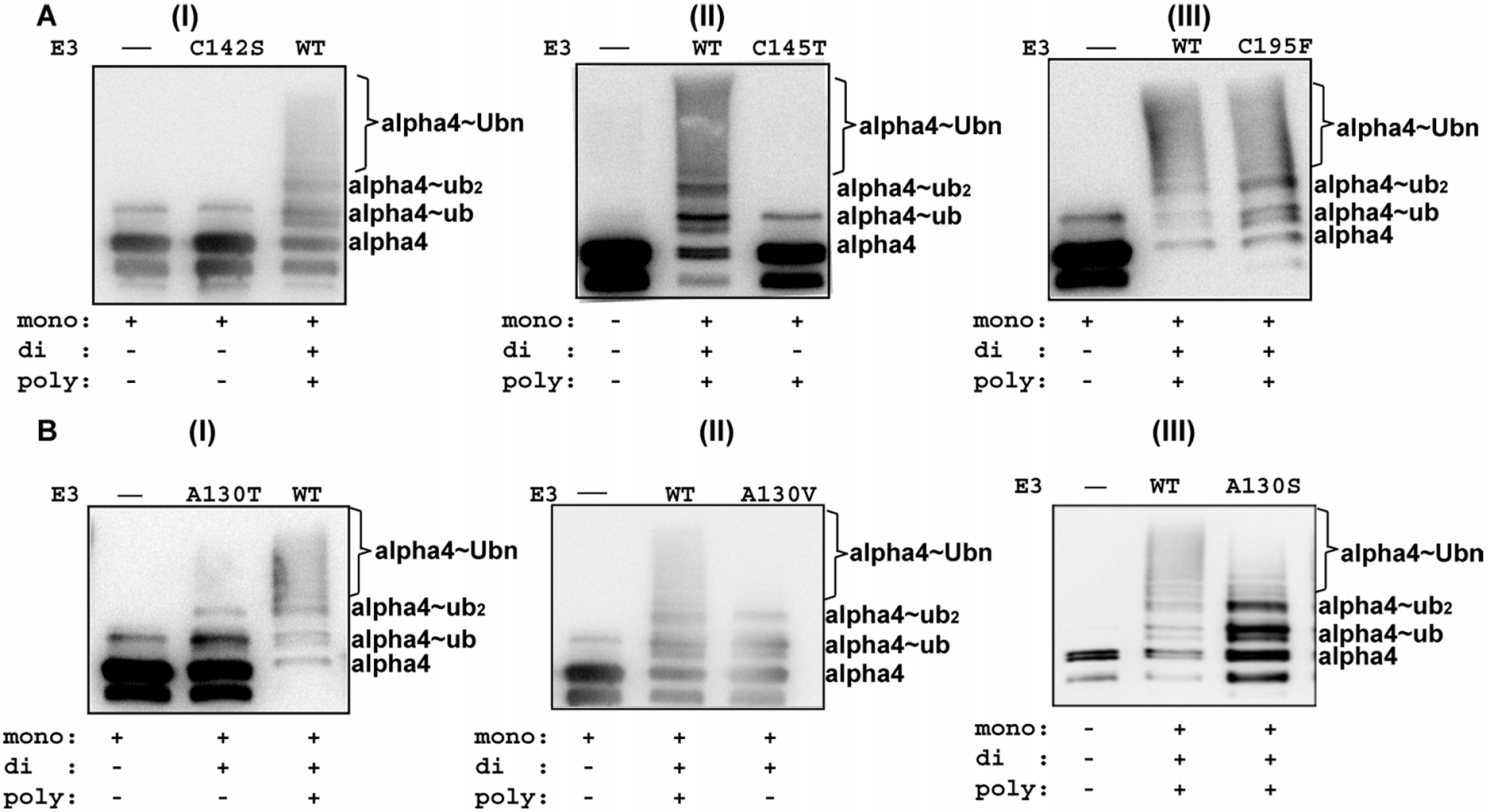
XLOS-observed mutations within Bbox1 disrupted polyubiquitination of alpha4. **A.** Ubiquitination assay of alpha4 was performed with the RB1B2 construct containing (i) C142S, (ii) C145T, and (iii) C195F. **B.** Ubiquitination of alpha4 by RB1B2 with the (i) A130T, (ii) A130V and (iii) A130S mutations within the Bbox1 domain. The amount of alpha4 used for the assay with the A130T RB1B2 mutant was higher than that used for the A130V mutant assay. Nonetheless, the overall results for the two RB1B2 mutants are very similar. For comparison, wild type RB1B2 was shown to catalyze polyubiquitination of alpha4, as indicated by the smear/ladder pattern designated with the labeled alpha4∼Ub_n_. Negative controls in which RB1B2 protein is omitted are shown for each reaction, as well. At the bottom of each figure, the ability of the mutant RB1B2 constructs to catalyze the ubiquitination of alpha4 is qualitatively noted. Antibody was specific for the C-terminal region of alpha4.

We performed the ubiquitination assays with full-length alpha4 using the RB1B2 mutants, and the wild type RB1B2 protein as control. The RB1B2 proteins harboring the C142S and C145T mutations were unable to catalyze the polyubiquitination of alpha4. Only a weak band corresponding to mono-ubiquitinated alpha4 was observed (Figure 5A(i, ii)). In contrast, the RB1B2 protein with the C195F mutation within the Bbox2 domain catalyzed the polyubiquitination of alpha4 (Figure 5A(iii)).

The RB1B2 construct containing the A130T or A130V mutations was unable to catalyze the polyubiquitination of alpha4 (Figure 5B(i, ii)). With both mutants, a strong mono-ubiquitinated alpha4 band was observed. A weak band corresponding to di-ubiquitinated alpha4 was also observed. Even though we added slightly greater amount of alpha4 in the ubiquitination assay with A130T (Figure 5B(i)), the similarity in results suggests that the mutations of A130 to threonine or valine might have similar structural and functional effects. The RB1B2 protein containing the A130S mutation was capable of catalyzing the polyubiquitination of alpha4. The intensity of the polyubiquitinated alpha4 bands was reduced when comparing the ratio of the mono- and di-ubiquitinated bands to the smear pattern for the mutant and wild type RB1B2 protein (Figure 5B(iii)).

## Discussion

In this study, we provide the first direct evidence that MID1 could catalyze the ubiquitin-modification of PP2Ac and therefore could regulate the function of PP2A (Figure 2A). Previous description of the regulation of PP2A included phosphorylation of the various subunits, carboxymethylation of PP2Ac, and association with other protein complexes, including inhibitors I_1_^PP2A^ and I_2_^PP2A^ (see reviews [43], [44]). Indication that PP2A could be regulated by ubiquitination was suggested from studies involving alpha4 and the protestin-inducible EDD (E3 identified by differential display) E3 ubiquitin ligase [45]. However, it is unclear whether EDD is directly involved in the ubiquitination of PP2Ac.

The role alpha4 plays in regulating the ubiquitination of PP2Ac by MID1 appears to be complex. On one hand, alpha4 is shown to be a potent regulator of PP2Ac [4], [46]. Alpha4 was shown to bind and induce a conformational change that inactivated the PP2Ac subunit, and this complex apparently protected the PP2Ac subunit from degradation [4], [21]. On the other hand, alpha4 is postulated to facilitate a MID1-alpha4-PP2Ac complex that promoted the ubiquitination of PP2Ac by MID1 [17], [24], [37]. Our ubiquitination data suggest that MID1 could interact with and target PP2Ac in the absence of alpha4 (Figure 2A). The mechanism associated with the reduction in the level of ubiquitination of PP2Ac in the presence of alpha4 is unclear. A first speculation for the reduction might be that MID1 catalyzes the ubiquitination of alpha4, decreasing the available ubiquitin [1]. However, the amount of ubiquitin present was at saturating concentration and sufficiently high to account for the polyubiquitination of both the PP2Ac and alpha4 proteins. Results of the titration of alpha4 from sub-molar equivalents to molar excess compared to the amount of PP2Ac protein present were inconclusive in confirming a diphasic dependence of alpha4 concentration, as was demonstrated for arrestin3 and JNK3α2 in phosphorylating MAP kinases [41]. Further studies are warranted to understand the mechanism of regulation.

Consistent with our previous studies, the RB1B2 protein construct of MID1 is sufficient for catalyzing the ubiquitination of PP2Ac (Figure 2C). It appears that C-terminal domains (CC, FNIII and SPRY/B30.2) may also be important for efficient targeting of PP2Ac. The Bbox domains are required for at least the diubiquitination of PP2Ac (Figure 2D). Without the Bbox domains, the RING domain only facilitated the mono-ubiquitination of PP2Ac. Even though the Bbox domains possess RING E3-like folds [47], [48] they are unable to catalyze the ubiquitination of PP2Ac, indicating that the Bbox domains may function, in part, as E4 ligases, similar to the roles of the RING domains of BARD1 and MDMX [49], [50], [51], [52].

**Figure 6:**
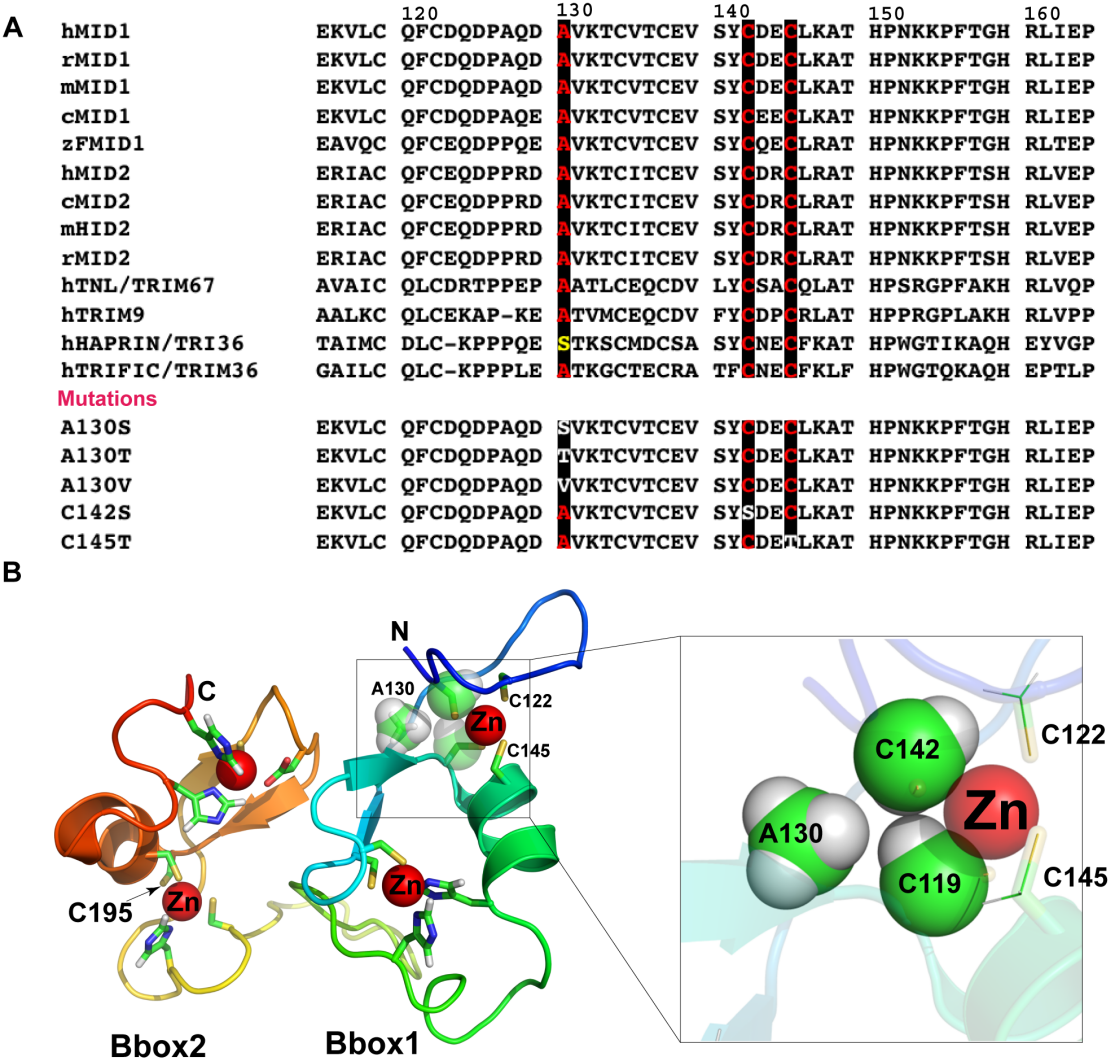
XLOS-observed mutations of highly conserved amino acids destabilized the Bbox structure. **A.** Sequence alignment of the MID1 and MID2 Bbox1 domains from human, rat, mouse, chicken and zebrafish. Conserved A130, C142 and C145 residues are highlighted in red font and dark shading. At the bottom, the sequences of the various Bbox1 mutations are shown. **B.** Ribbon representation of the tertiary structure of the Bbox1-Bbox2 domains in tandem showing the coordinated zinc ions (red spheres) and their ligands. The structure shows the locations of the amino acids that were mutated, specifically residues Ala130, Cys142, Cys145 and Cys195. On the right, a zoomed view showing the relative proximity of Ala130 to the zinc binding Cys119 and Cys142 residues. Space filling representation of the side chain atoms of these three amino acids suggests that mutation of Ala130 to Thr and Val will induce steric clashes with the side chain atoms of Cys119 and Cys142, which can disrupt zinc-coordination.

In addition to understanding how natural mutations within the Bbox domains of MID1 affect its E3 ligase activity, these mutations also allows us to gain insights into the mechanism of Bbox domain function. The Cys142 and Cys145 residues are highly conserved (Figure 6A) in Bbox1 domains throughout TRIM proteins. Mutations of these zinc-binding cysteine residues to serine or threonine, residues typically not associated with coordinating zinc ions, should disrupt the coordination of the zinc ion and cause significant structural changes of the Bbox1 domain [48], [53], [54], [55], [56]. Similarly, the C195F mutation should disrupt the structure of the Bbox2 domain.

Residue Ala130 is also highly conserved in Bbox1 domains (Figure 6A). Even thought Ala130 is not involved in metal coordination the structure of the Bbox1 domain shows the side chain methyl group in close proximity to methylene atoms of Cys119 and Cys142 (Figure 6B). We postulate that the larger side chains of valine or threonine would steric hinder the position of side chain of Cys119 and Cys142 and their coordination of the zinc ion. Thus, the A130T/V mutation could have similar structural effects as the mutations of the Cys142 and Cys145 residues. In contrast, the A130S mutation is equated to replacing of one of the methyl protons of alanine with a hydroxyl moiety. The hydroxyl group is flexible and provides less steric clashes with the cysteine side chains, allowing the Bbox1 domain to maintain native function.

Perturbation of the Bbox1 domain structure could disrupt the interaction of RB1B2 protein with the alpha4 protein. In a non-biased functional screen, we previously identified a L146Q substitution that abolished the MID1-alpha4 interaction and the subsequent ubiquitination of alpha4 [1]. Thus, the loss of polyubiquitination of alpha4 indicates that the A130T/V, C142S and C145T mutations destabilize the interaction necessary for ubiquitination of alpha4. In these instances, the Bbox2 domain was unable to compensate for the loss of function of the Bbox1 domain.

Qualitatively, the mutations within the Bbox1 domain did not affect the ability of the RB1B2 protein to catalyze the di-ubiquitination of PP2Ac. Given that the both RING domain and folded Bbox1 domain are required for catalyzing the diubiquitination of PP2Ac (Figure 2D), the diubiquitination of PP2Ac by the RB1B2 with mutations within the Bbox1 domain suggests that the Bbox2 domain compensated for the loss of function of the mutant Bbox1 domain, at least in the targeting PP2Ac.

Interestingly, the RB1B2 protein with the C195F mutation was observed to catalyze the ubiquitination of PP2Ac and alpha4 (Figures 4, 5B). These results were somewhat unexpected because the C195F mutation is associated with XLOS and should have an effect on the targeting of PP2Ac or alpha4. Thus, it is possible that the Bbox2 domain may have other roles that could not be distinguished from the current studies.

It is intriguing to note that the different mutations within the Bbox1 domain, which should have resulted in same loss of structure and function, do not correlate with the same set of birth defects (Figure 1B). The truncation MID1 mutation that yields just the RING-Bbox1 domains (Figures 1A and B) is associated with overlapping defects as Bbox1 domain mutations. Similarly, the C195F mutation is associated with overlapping birth defects as the A130T/V mutations, with two additional defects (anal malformation and developmental delays) (Figure 1B). These results suggest that there may be other factors that contribute to XLOS, one of which may be the role the highly homologous MID2 [38].

In conclusion, MID1 could regulate the function of PP2A by catalyzing the ubiquitination PP2Ac and alpha4. It is possible that many of XLOS-observed mutations, including mutations of the Bbox1 domain, result in an increase of unmodified or monoubiquitinated alpha4, which could subsequently bind and protect PP2Ac from ubiquitin-mediated degradation. Thus, alpha4 may have a significantly greater role in contributing to X-linked Opitz syndrome that includes protecting PP2Ac.
